# Bifenthrin Residues in Table Grapevine: Method Optimization, Dissipation and Removal of Residues in Grapes and Grape Leaves

**DOI:** 10.3390/plants13121695

**Published:** 2024-06-19

**Authors:** Saleh S. Alhewairini, Rania M. Abd El-Hamid, Nevein S. Ahmed, Sherif B. Abdel Ghani, Osama I. Abdallah

**Affiliations:** 1 Department of Plant Protection, College of Agriculture and Food, Qassim University, P.O. Box 6622, Buraydah 51452, Saudi Arabia; sherifbiomy@yahoo.com; 2 Department of Pesticide Residues and Environmental Pollution, Central Agricultural Pesticide Laboratory, Agricultural Research Center, Giza 12618, Egypt; ranianana82@yahoo.com (R.M.A.E.-H.); dr.nevein@gmail.com (N.S.A.); 3 Department of Plant Protection, Faculty of Agriculture, Ain Shams University, P.O. Box 68 Hadayek Shoubra, Cairo 11241, Egypt

**Keywords:** bifenthrin pesticide, method validation, dissipation, grapes, pesticide residues removal

## Abstract

The QuEChERS method was adjusted to determine bifenthrin residues in grapes and grape leaves. Extraction and cleanup procedures were optimized to decrease co-extracted materials and enhance the detection of bifenthrin. The method was validated per the European Union (EU) Guidelines criteria. Accuracy ranged from 98.8% to 93.5% for grapes and grape leaves, respectively. Precision values were 5.5 and 6.4 (RSDr) and 7.4 and 6.7 (RSD_R_) for grapes and grape leaves, respectively. LOQs (the lowest spiking level) were 2 and 20 µg/kg for grapes and grape leaves, respectively. Linearity as determination coefficient (R^2^) values were 0.9997 and 0.9964 for grapes and grape leaves, respectively, in a matrix over 1–100 µg/L range of analyte concentration. This was very close to the value in the pure solvent (0.9999), showing the efficiency of the cleanup in removing the co-extracted and co-injected materials; the matrix effect was close to zero in both sample matrices. Dissipation of bifenthrin was studied in a supervised trial conducted in a grapevine field during the summer of 2023 at the recommended dose and double the dose. Dissipation factor k values were 0.1549 and 0.1672 (recommended dose) and 0.235 and 0.208 (double dose) for grapes and grape leaves, respectively. Pre-harvest interval (PHI) was calculated for the Maximum Residue Limit (MRL) values of the EU database. Residues of bifenthrin were removed effectively from grapes using simple washing with tap water in a laboratory study. Residues reached the MRL level of 0.3 mg/kg in both washing treatments, running or soaking in tap water treatments for 5 min. Removal from leaves did not decrease residue levels to the MRL in grape leaves.

## 1. Introduction

Grapevine (*Vitis vinifera* L.) is indigenous to the Middle East. It is cultivated for its fruit which is consumed fresh, processed, or as derivative products. Grape leaves are also used in Mediterranean cuisine. Egypt was ranked the fourth table grape producer worldwide in 2022, with a total production of about 1.6 M metric tons of grapes, and the fifth exporter of vine leaves, with an export value of 169 million Dollars [1].

Grapes are susceptible to infestation by different insect pests such as mealybugs, fruit flies, and thrips. As the Ministry of Agriculture of Egypt recommends, Bifenthrin is frequently used to control these pests’ infestations in grapes.

Bifenthrin [2-methylbiphenyl-3-ylmethyl (Z)-(1RS)-*cis*-3-(2-chloro-3,3,3-trifluoroprop-1-enyl)-2,2-dimethyl cyclopropanecarboxylate] is a non-cyano pyrethroid type pesticide with a cyclopropyl ring and belongs to the photo-stable third-generation synthetic pyrethroids [2]. It is classified as type II in terms of its mammalian toxicity, according to the World Health Organization (WHO). Bifenthrin temporarily binds to voltage-gated sodium channels in insects and mammals, delays the achievement of rest status of neurons, and causes continuous action potential of axons and eventually paralysis [3]. Bifenthrin is a non-systemic insecticide/acaricide used to control several agricultural pests and disease vectors [4].

Bifenthrin residues in food could present potential risks to humans. Bifenthrin has cytotoxic and genotoxic effects on human epithelial cell lines [5], neurotoxicity and hepatotoxicity [6], oxidative stress, inflammation, and neuron degradation [7,8]. Bifenthrin is categorized as an endocrine-disrupting pollutant [9].

Several studies reported residues and dissipation of bifenthrin in different crops, including palm dates [9], peas [10], green beans [11], pears [12], and tea [13].

Studying the dissipation behavior of pesticide residues in crops is essential for estimating Pre-Harvest Intervals (PHI) of pesticides in treated crops. PHI is the time, after application, required to attenuate specific pesticide residues to the level of or less than the maximum residue limit (MRL) set by legislative bodies.

Bifenthrin was analyzed using gas chromatography (GC) equipped with an electron capture detector (µECD) [14] or coupled to mass spectrometry (GC-MS, GC tandem mass spectrometry (MS/MS)) [9,15,16] in agricultural commodities. Liquid chromatography coupled to mass spectrometry (LC-MS/MS) is less used to determine bifenthrin and pyrethroids in general [11,17]. However, electrospray ionization–mass spectrometry showed higher sensitivity and lower limit of detection (LOD) [17].

The present study was undertaken to develop and validate a simple and sensitive method for quantification of bifenthrin residues in grapes and grape leaves using modified QuEChERS (quick, easy, cheap, effective, rugged, and safe) method employing liquid chromatography with tandem mass spectrometry (LC-MS/MS) with particular focus on the cleanup step. In addition, the developed method was utilized to determine the dissipation behavior of bifenthrin in its oil-in-water (EW) formulation in the vineyard (table grape, Seedless Thompson) in grapes and leaves and to estimate Pre-Harvest Interval (PHI). In addition, studying the removal of bifenthrin residues from grapes and leaves using simple washing treatments with tap water.

## 2. Results

### 2.1. Optimization of LC-MS/MS

**Figure 1:**
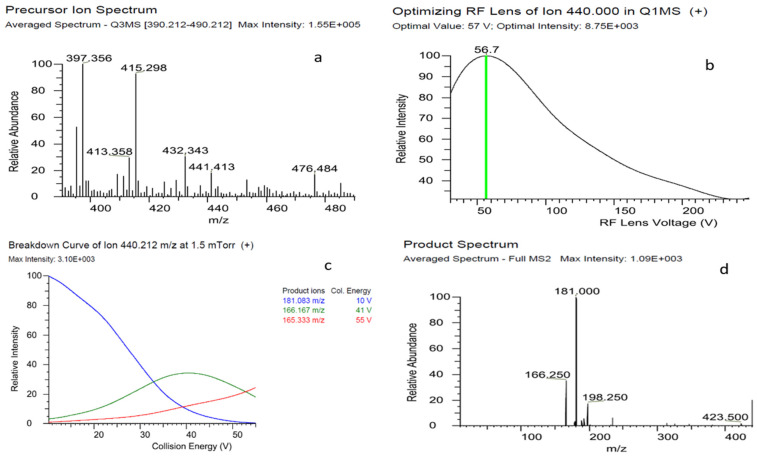
Full scan spectrum (**a**), optimized Rf Lens (**b**), breakdown curve at 1.5 mTorr (+) (**c**), and product spectrum (**d**) of bifenthrin.

The optimization of mass spectrometer parameters to determine bifenthrin was carried out meticulously (Figure 1). A standard bifenthrin solution at a concentration of 1 mg/L was infused in a 50:50 *v*/*v* mixture of water and methanol. Bifenthrin did not show ionization in negative electrospray ionization (ESI^−^) mode. Different mobile phase compositions were tested to enhance ionization and detection of bifenthrin, i.e., formic acid and ammonium formate. Acidic medium enhanced the ionization of bifenthrin through adduct ion formation [10]. The presence of ester moiety in pyrethroids allows the formation and stabilization of such adducts [18]. The lone pair of electrons of non-carbonyl oxygen of the ester group creates a double bond with the carbonyl carbon that drives the π bond electrons to move to the carbonyl oxygen, producing a negative charge on it, which is required for the abduction of Na^+^ or NH_4_^+^ ion from the solution. The bifenthrin–ammonium adduct [M+NH_4_]^+^ ion was more abundant than the protonated bifenthrin [M+H]^+^ ion. This might be attributed to the fact that H^+^ has more affinity to form a covalent bond with a formate base in the mobile phase than the carbonyl oxygen of bifenthrin. Bifenthrin and several other pyrethroids produced stable sodium adducts [M+Na]^+^ [19,20]. In total, 5 mM ammonium formate was integrated into the mobile phase. The infusion flow rate of the syringe pump was precisely set to 5 µL/min with a mobile phase flow rate of 0.3 mL/min. Using the Q1 scan type was crucial for identifying the peak signal intensity for the molecular ion. Subsequent MRM scans were used to determine the most intense and selective precursor/product ion pair while determining the optimal values for ion optics. The collision energy (CE) voltage was optimized to 41 V for the quantification ion (181 *m*/*z*) and 10 V for the qualification ion (166.2 *m*/*z*), with the RF lens voltage set to 56.7 V (Figure 1). The retention time (R_t_) was 9.07 min., bifenthrin–ammonium ion adduct was utilized as the molecular ion [M+NH_4_]^+^ of 440.1 *m*/*z*, and product ions were 181 and 166.2 *m*/*z*. Using these optimized parameters ensured maximum ion transfer and, thus, significantly increased sensitivity.

Due to its complexity, the final extract from grape leaves was selected for further testing to optimize chromatographic separation. Blank samples of grape leaves were collected from the local market and were pre-analyzed using the AOAC QuEChERS method [21] to ensure the absence of the target analyte.

The separation of bifenthrin was first tested using a mobile phase of acetonitrile/water and methanol/water with 5 mM ammonium formate at a gradient elution, a flow rate of 0.3 mL/min, and a column temperature of 40 °C. When methanol/water with 5 mM ammonium formate was used, a symmetrical peak shape and a higher response were obtained compared to acetonitrile/water. The addition of formic acid (0.1%) to the mobile phase reduced the peak area by about 48%.

### 2.2. Optimization of Cleanup

**Figure 2:**
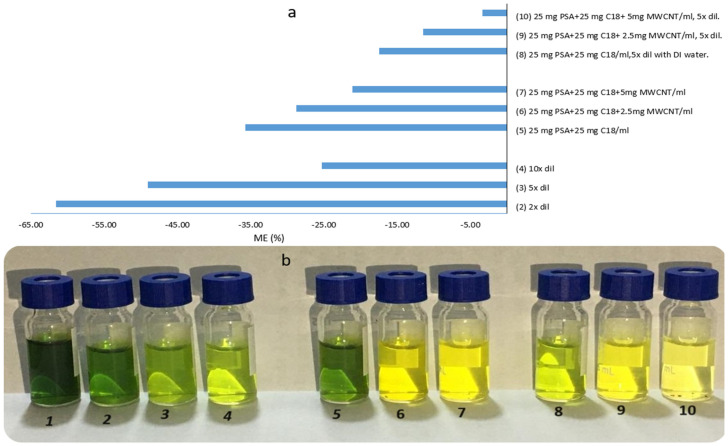
(**a**) The efficiency of various adsorbents and dilution ratios in grape leaf extract cleanup at 0.1 mg/kg (n = 5). (**b**) Raw extract of grape leaves (1), 2x dil (2), 5x dil (3), 10x dil (4), PSA + C18 (5), PSA + C18 + 2.5 MWCNTs (6), PSA + C18 + 5 MWCNTs (7), PSA + C18 + 5x dil (8), PSA + C18 + 2.5 MWCNTs + 5x dil (9), and PSA + C18 + 5 MWCNTs + 5x dil (10).

In this study, the cleanup step was refined by testing different combinations of adsorbents, including primary, secondary amine (PSA), octadecyl (C18), and multi-walled carbon nanotubes (MWCNTs), to evaluate their impact on reducing matrix effects during analysis. A mixture of 25 mg PSA, 25 mg C18, and 150 mg MgSO_4_ per 1 mL extract was tested individually and analyzed after adding 2.5, 5, and 10 mg MWCNTs. Dilution tests of the raw and adsorbent-cleaned extract obtained using the QuEChERS method with DI water were performed. The extracts were diluted 2-fold, 5-fold, and 10-fold. The dilution rates were evaluated based on the matrix effect for each dilution (Figure 2). The matrix effect was determined by comparing the areas of the analytical standard of bifenthrin in the solvent and the solutions prepared in a matrix-matched extract, as described by Pucci et al. [22]. Figure 2 shows the graph of the matrix effect for the different adsorbents and dilution rates tested and the color change in extracts as it appears in crude and clean extracts. Values between −20 and +20% are not considered a significant matrix effect as this variation is close to the repeatability values. Values between −50 and −20% or +20 and +50% represent a medium matrix effect, and values below −50% and above +50% indicate a strong matrix effect [23]. The results show that a 10-fold dilution reduces the amount of interfering substances in the extract. However, the matrix effect was still moderate, with an average value of −25%. This dilution rate corresponds to a 20-fold sample dilution, considering that initially 5 g was extracted with 10 mL acetonitrile. This dilution could reduce the final quantified amount and the LOQ (the lowest spiking level). In addition, using 10 mg MWCNTs significantly removed the co-extracts, but this also significantly affected the recovery rate of bifenthrin, which reached only 62%. A 2-fold and 5-fold dilution significantly reduced the interfering compounds compared to the raw extract, with matrix effect rates of −61% and −49%, indicating a high and moderate suppression effect, respectively. As for the use of adsorbents, the use of 25 mg of PSA plus 25 mg of C18 and their use in the same amount after the addition of 2.5 mg and 5 mg of MWCNTs resulted in a reduction in co-extractants with matrix effect rates of −35%, −28%, and −21%, respectively, which are all moderate effects. Thus, the mixture of 25 mg PSA, 25 mg C18, and 5 mg MWCNTs with 150 mg MgSO_4_, followed by a 5-fold dilution process, was employed, which showed the weakest suppression effect of −3.36%.

### 2.3. Method Validation

Linearity and matrix effect: A linear dynamic range was achieved between 1 and 100 µg/L, corresponding to 1 and 100 µg/kg in grape samples and 10 and 1000 µg/kg in grape leaves, with six calibration points. Linearity as the coefficient of determination (R^2^) was calculated from the calibration curves of standard in pure solvent and standard in a matrix of grapes and grape leaves (Table 1). The R^2^ value was 0.9999 for standard in pure solvent, compared to 0.9997 and 0.9964 in grapes and grape leaves final cleaned extract, respectively, showing a good fit of the representative line model to the actual data.

Matrix effect: The matrix effect in LC/MS-MS is the effect of co-eluting sample components on measurement results due to either suppression or enhancement of ionization of analytes [24]. Matrices with a high percentage of dry matter show higher matrix effects than those with high water-content matrices. Also, samples with high pigment content have similar effects. Using matrix-matched standards for quantification is an effective solution to compensate for the expected inaccuracy in measurement. However, the calculated matrix effect should be within −20 to +20 [9]. Cleanup or sample dilution steps may reduce co-extracted and co-injected materials [23]. Matrix effects were quantitatively assessed for grape and grape leaf samples using the optimized adsorbents mixture of 25 mg PSA, 25 mg C18, and 150 mg MgSO_4_ per 1 mL extract (for grape), in addition to 5 mg of MWCNTs and 5 times dilution for grape leaves. The matrix effect observed in grapes was −1.17% and −3.14% in grape and grapes leaves, respectively, indicating a slight suppression effect on the ionization efficiency of the bifenthrin (Table 1). Based on the criteria of the SANTE guidelines [25], this level of suppression is considered negligible.

#### 2.3.1. Accuracy

The method accuracy was calculated as the average recovery% at all tested spiking levels for grapes or grape leaves (Table 1). Recovery% (n = 6 replicates) ranged from 97.7 to 100.7 for grapes and 90.9 to 96.4 for grape leaves, with an average recovery percentage of 98.8 and 93.5 for grapes and grape leaves (Table S1), respectively, which are following the EU regulations [25], which stipulate that the accepted recovery rate should range between 70 and 120%.

#### 2.3.2. Precision

Precision was calculated as the relative standard deviation RSD within the same day (intraday repeatability RSDr) and between three different days (interday repeatability RSDR) at 2 µg/kg for grapes and 20 µg/kg for grape leaves (Table 1). RSDr was calculated from recovery experiments replicates (six replicates) performed on the same day. RSDR was calculated from recovery experiment replicates (18 replicates) performed on three different days with 7 day intervals. RSDr values were 5.5 and 6.4, and RSDR values were 7.4 and 6.7 for grapes and grape leaves, respectively. Obtained precision values were in the accepted norms (<20%) of the EU regulations [25].

#### 2.3.3. LOD and LOQ

The LOD and LOQ were calculated as the values of 3-fold and 10-fold of the background signal of the grape and grape leaf matrix, respectively. LOD values were 0.48 and 0.42 µg/kg, and LOQs (the lowest spiking levels) were 2 and 20 µg/kg for grapes and grape leaves, respectively (Table 1), considering the dilution factor of 10 times in grape leaves. The recovery tests were conducted at these levels for confirmation and achieved an average recovery of 92.6% and 91.1%, respectively.

The QuEChERS method was implemented in several previous studies with modifications in the cleanup step and the use of GC-MS or GC-MS/MS instead of LC-MS/MS, which was used in this study. Abdel Ghani et al. (2018) developed dispersive liquid–liquid micro-extraction (DLLME) in combination with GC-MS for the determination of bifenthrin in palm dates after extraction with acidified acetonitrile; the achieved validation criteria were 86–101.3%, 0.79 µg/kg, +12.27%, for recovery percentage, LOQ, and ME% [9]. LC-MS/MS was recently used to determine bifenthrin in different matrices [11,26] and revealed a strong matrix suppression effect ranging from −38.6% to −80.4%, significantly higher than our findings of −1.17% and −3.4% in grape and grape leaves, respectively. GC-MS and GC-MS/MS were more commonly used in most published research, and the LOQ value for these methods depended on the protocol used to prepare the samples. In this study, the LOQ, accuracy, precision, and ME% were satisfactory enough to determine bifenthrin using the proposed extraction and cleanup procedure in conjugation with LC-MS/MS.

### 2.4. Dissipation Kinetics

Dissipation of bifenthrin in grapevine, including grapes and leaves, was studied using the developed method. The residual amount of bifenthrin was followed for 28 days after application at the recommended and double doses. Initial deposits in the case of leaves were about 30 times more than grapes in both recommended and double dose treatments due to weight to surface area ratio. Abdel Ghani and Abdallah obtained about 40 times higher initial deposit of chlorfenapyr in the leaves of okra and squash than the corresponding fruits [27]. Motorized knapsack Air-assisted mist blower sprayer caused 10 times more bifenthrin deposits in green peas’ leaves than in the flattened fruit pods [10]. The application of a double dose caused a 60% extra deposit than the recommended amount in the grape and leaf samples. Double-dose treatment of chlorfenapyr caused from 48% to 80% additional deposits in okra and squash leaves and fruit [27]. Dissipation regression curves were plotted using natural logarithm (ln) of residues data (mg/kg) against time (days). Dissipation rate (k) values were 0.1549 and 0.1672 days^−1^ for grapes and 0.235 and 0.208 days^−1^ for grape leaves, respectively, at the recommended dose and double dose. Half-life (t_1/2_) values were 4.08 and 3.54 days for grapes and 1.885 and 2.14 days for grape leaves, respectively, at the recommended and double doses. Bifenthrin half-life values were from 30 to 33 days in kumquat fruit with initial deposits of 0.145 to 0.198 mg/kg using a 10% emulsifiable concentrate (EC) formulation at 60 mg/L spray solution, and the application method was not mentioned [26]. Leaves showed a faster dissipation rate than grapes in both doses, appearing in k and t_1/2_ values. Leaves receive more bifenthrin spray than grapes and are more exposed to environmental factors such as sunlight. In green tea leaves, initial deposit values were from 11.4 mg/kg to 26.6 mg/kg, and half-life value of about 1 day [28]. The dissipation rate followed first-order kinetics in both grapes and leaves. Determination coefficient (R^2^) values were more than 0.987 proving good fitness of the regression line model (first-order) with the found residue data. PHI values were 6 or 8 for grapes and 32 or 36 days for leaves at normal and double doses, respectively. PHI of bifenthrin was 4 days in peas using 2.5% EC bifenthrin [10]. PHI reached 42 days in palm date fruit at an MRL level of 0.01 mg/kg [9]. PHI values were increased by 30% in grapes and about 10% in leaves at the double dose treatment. A 50% increase in PHI value was observed in the double-dose treatment of bifenthrin in palm dates [15]. However, using a 2% wettable powder (WP) formulation in pear produced no residues after 7 days of application [12]. Table 2 shows dissipation parameters and PHI of bifenthrin in grape and grape leaves. Residues of bifenthrin found in filed samples are tabulated in Table S2.

### 2.5. Removal of Bifenthrin Residues by Washing

Washing with water is the easiest, non-toxic, effective, and most accessible household approach to remove pesticide residues from fruits and vegetables [29]. The removal efficiency depends on several factors, including the pesticide dose, its formulation type and solubility in water formulation, and vegetable surface area and roughness [30]. Bifenthrin has a log *p* value of 6.0 and exhibits very low water solubility (0.1 mg/L); in that case, removal efficiency will only depend on formulation type, treated surface area, and roughness of the surface. In the case of leaves, the ratio of surface area to weight is high, meaning more reception of residue doses, and the surface is rough, which impedes the removal process. On the contrary, the grapes’ surface is smooth, facilitating the removal of residues, and the surface-to-weight ratio is low, making it possible to reach legitimate levels of residues.

Two simple washing treatments, washing with running tap water and soaking in tap water, were performed at two different times, i.e., 1 and 5 min. The initial residue concentration was 0.6 mg/kg of bifenthrin.

For grapes, the effect of the four treatments was significantly different from the untreated control (*p* ≤ 0.05) (Table 3). Increasing treatment time significantly increased the washing effect (*p* ≤ 0.05). Both 5 min treatments caused a 50% reduction in efficiency, and residues reached 0.3 mg/kg concentration, the MRL level of bifenthrin in grapes. Applying pesticides and harvesting before required PHI is possible for some producers, especially producers targetting local markets that do not have control checks for pesticide residue content. So, this simple treatment decreased the residues to the MRL level, reducing the potential risk to the unaware consumer. Aqueous acetic acid solutions removed 23.3% to 26.6% of carbofuran from treated potatoes [31]. Washing with tap water for 3 min caused a reduction of 40% in tomato and 65% in pepper for the low water-soluble fungicide boscalid [29]. Also, washing with tap water for 3 min reduced 26% of chlorpyrifos (water solubility 1.4 mg/L) in nectarine with similar deposited residues of the current study (5.9 mg/kg) [31]. Dimethomorph residues in pepper fruit were removed by about 32% by rinsing three times in water [31]. Only 10% of etoxazole was removed from orange fruit using water washing treatment, which might be attributed to the rough surface of the orange and the very low residue deposit of etoxazole [31].

Concerning grape leaves, the initial concentration of bifenthrin was 10.4 mg/kg. Statistical analysis showed that all treatments decreased bifenthrin residues and significantly differed from the untreated control (*p* ≤ 0.05). Also, 5 min treatments in running water or soaking were more effective in reducing bifenthrin residues than 1 min treatments. However, all treatments failed to decrease bifenthrin residues to the MRL level of bifenthrin in grape leaves (0.01 mg/kg) [32]. The removal of fenvalerate (log *p* 6.2) and dicofol (log *p* 4.3) from basil leaves using tap water for 15 min was moderately efficient, among other chemical treatments [31]. However, Sung-Woo and coworkers reported that dimethomorph residues showed an 84% reduction in pepper leaves by rinsing three times in water [33], which might be due to the smooth surface nature of pepper leaves, unlike the very rough grape leaves. Other washing treatments should be sought to remove bifenthrin in grape leaves. The utmost limitation to evaluate the efficiency of a treatment to remove bifenthrin residues is the very low MRL value of bifenthrin (0.01 mg/kg); this value is suggested to be revisited by the EU. The MRL values of chlorantraniliprole are 1 mg/kg and 20 mg/kg in grapes and grape leaves, respectively. The value of leaves is 20 times that of grapes, which is rational according to the surface-to-weight ratio concern, which is not the case in corresponding bifenthrin MRL values.

## 3. Materials and Methods

### 3.1. Chemicals

Reference standard bifenthrin (98.8% purity) was purchased from Chem Service Inc. (West Chester, PA, USA). HPLC-grade acetonitrile and glacial acetic acid, LC-MS grade methanol, formic acid, and ammonium formate were purchased from Fisher Scientific (Loughborough, UK). Primary Secondary Amine (PSA) and octadecyl-modified silica (C18) were purchased from Agilent Technologies Inc. (Wilmington, DE, USA). Multi-Walled Carbon Nanotube (MWCNT) was purchased from Shilpent Co. (Shilpa Enterprises, Maharashtra, India). Anhydrous magnesium sulfate and sodium acetate were purchased from Chem-Lab NV (Zedelgem, Belgium). The ultrapure deionized water (DI) was produced using the Evoqua Ultra Clear system (Evoqua Water Technologies LLC., Guenzburg, Germany). Baldario formulation (Bifenthrin, 10% EW), a Shandong United Pesticide Company Ltd. (Taian, China), China product, was purchased from the local market.

### 3.2. Standard Solutions

A stock standard solution of 1000 mg/L of bifenthrin was prepared in MeCN. A working standard solution (WS) of 10 mg/L was prepared freshly by diluting the stock solution in MeCN and used for further dilutions. Dilutions used for the standard calibration curve in solvent (SS), i.e., 0.5, 1.0, 2.5, 5, 10, 25, 50, 100, and 250 µg/L, were prepared in MeCN. As in the case of SS, matrix-matched standard (MMS) calibration solutions were made using the corresponding extract of blank samples instead of MeCN. MMS was utilized in calculations of matrix effect and residue determination of actual samples generated from field or laboratory experiments. Stock solutions were stored at −20 °C; SS, WS, and MMS were stored at +4 °C. Where required, the extracts of real samples with high concentrations were diluted using blank extracts.

### 3.3. Sample Preparation, Extraction, and Cleanup

In total, 10 ± 0.2 g of frozen homogenized grapes or 5 ± 0.1 g of frozen homogenized grape leaves were weighed in a 50 mL centrifuge tube; 10 mL of acidified acetonitrile (1% acetic acid) was added to the homogenized grapes; 5 mL of DI water was added to grape leaves and shaken for 1 min, and then 10 mL of acidified acetonitrile was added. Tubes were vortexed for 3 min at high speed with an inserted ceramic homogenizer. Premixed 4 g of magnesium sulfate and 1 g sodium acetate were added to each tube and hand-shaken for 30 s before centrifugation at 5000 rpm for 5 min. In total, 2 mL of the upper supernatant was cleaned up in a 2 mL centrifuge tube using 300 mg MgSO_4_, 50 mg PSA, 50 mg C18 for grape samples, and 300 mg MgSO_4_,50 mg PSA, 50 mg C18, 5 mg MWCNT for grape leaves in dispersive solid phase micro-extraction SPME fashion, vortexed for 1 min, and then centrifuged at 5000 rpm for 5 min. The cleaned extract was filtered through a 0.22-micron nylon syringe filter into an LC vial (for grape), and the cleaned leaf extract was diluted 5 times in acetonitrile/water (1:1) before filtration into an LC vial for quantitation analysis.

### 3.4. LC-MS/MS

A Dionex Ultimate 3000 RS UHPLC module Liquid Chromatograph (LC) system (Thermo Fisher Scientific, Austin, TX, USA) equipped with TSQ Altis triple quadrupole mass spectrometer (Thermo Fisher Scientific, Austin, TX, USA) was used to perform the LC-MS/MS analysis. The chromatographic separation was performed on the Accucore RP-MS C18 column (100 × 2.1 mm, 2.6 m film thickness, Thermo Fisher Scientific) at 40 °C. The mobile phase consisted of (A) water and (B) methanol/water (98:2), both containing 5 mM of ammonium formate. The elution program started at 10% B, held for 1 min, and increased to 100% B over 3 min, where it was held for 10 min. After that, the percentage of B was decreased to 10% over 1 min, and it was held for 9 min in this condition, making a total run of 20 min.

Bifenthrin was detected using the multiple reaction monitoring (MRM) mode. The optimal MRM transitions were optimized in a positive electrospray ionization (ESI^+^) mode at a capillary voltage of 3.8 kV. Sheath and Aux gas were set at 40 and 10 Arb, respectively. The vaporizer and ion transfer tube were set at 350 and 325 °C, respectively. High-purity nitrogen was used as a nebulizer gas. Trace Finder software (version 4.1) was applied to acquire and process the obtained data.

### 3.5. Method Validation

Validation parameters, i.e., linearity, accuracy, precision, matrix effect, LOD, and LOQ (the lowest spiking level), were studied in accordance with the SANTE guidelines [24]. Linearity as R^2^ was assessed using a matrix-matched standard curve by plotting the obtained area against the corresponding concentration, i.e., 0.5, 1.0, 2.5, 5, 10, 25, 50, 100, and 250 µg/L (three replicates), using Microsoft Office Excel.

The accuracy of the method was calculated as the average recovery of experiments performed in five replicates at three levels (10, 100, and 1000 µg/kg) and (50, 100, and 1000 µg/kg) for grapes and grape leaves, respectively, on the same day and was repeated on different days. Recovery experiments were carried out by spiking 10 g of grape or 5 g of grape leaves (untreated blank samples) in a 50 mL centrifuge tube with a standard solution, and then mixing with a spatula and left to stand for 20 min. Spiked samples were extracted and determined using the same procedures for sample preparation, extraction, and cleanup. Recovery was calculated as the percentage of the obtained area against the obtained area of spiked matrix-matched extract at the same concentrations. The limit of detection (LOD) and quantitation (LOQ, the lowest spiking level) were calculated as 3:1 and 10:1 S/N ratios, and a recovery test was conducted to confirm the LOQ value. The method’s precision was calculated as the average of the relative standard deviation (RSD) of recovery experiment data at LOQ levels of grapes and grape leaves analyzed on the same day (intraday repeatability) and on three different days with 7 day intervals (interday repeatability) sextuplicate per day.

The matrix effect was calculated using the slopes of constructed matrix-matched standard and standard in solvent curves using Equation (1) [34].

ME = ((slope of MMS)/(slope of SS) − 1) × 100 (1)

### 3.6. Field Trial

A field trial was conducted in a grapeyard of table seedless grapes (Thompson), 30.63° N 31.94° E, El Salheya El Gedida, El Sharqia Governorate, Egypt. The experiments were carried out in June 2023. The average temperature was 30 °C (43 °C as maximum), relative humidity was 45–60%, and daylight was about 14 h during the experiment. The experimental area was split into three plots (150 m^2^ each). Baldario (Bifenthrin 10% EW) at 10 g and 20 g of active ingredient (a.i) per 100 L spray solution was applied using a 20 L motorized air-assisted mist blower sprayer (Cifarelli, Italy). Uniform coverage was achieved. Samples were collected at zero (3 h), 1, 3, 7, 10, 14, 20, 24, and 28 days after application. Following the European Commission guidelines [18,35], 12 primary samples were collected from each plot from 4 vines. Samples were taken from all sides of the vine and all levels. Heavily laden vines were the subject of more samples. Primary samples were combined in a bulk sample (≥4 kg), of which one kg was taken to the laboratory in a paper bag. Untreated samples for matrix effect study and method specificity were collected before application. Samples were comminuted and frozen at −20 °C until analyzed.

### 3.7. Removal of Bifenthrin Residues by Washing

Grapes and grape leaves (blank samples) were collected before pesticide application, samples of which were analyzed, and no trace of bifenthrin was found. Entire grape bunches and leaves were sprayed with Baldario (bifenthrin, 10% EW) solution. Treated units were taken and left to dry for 24 h. A representative sample (500 g) was taken to determine the initial residue level, and the rest was used for the washing treatments.

The washing treatments were (1) washing samples (500 g) using running tap water (10 L) for 1 min; (2) washing using running tap water for 5 min (the running rate of the tap water was controlled at 100 mL/second and temperature of 25 °C, and the distance between the samples and the running water source was 30 cm); (3) soaking of 500 g of sample in tap water (10 L, 25 °C) for 1 min; and (4) soaking 500 g of samples in tap water (10 L, 25 °C) for 5 min. After soaking treatments, grapes or grape leaves were rinsed for 30 s with tap water (100 mL/s, 25 °C) with hand rotation. All treatments were performed in triplicates. Samples were comminuted and frozen at −20 °C until analysis.

### 3.8. Statistical Analysis and Calculations

One-way analysis of variance (ANOVA) and *t*-test (two-tailed in pairs) were performed using IBM SPSS Statistics version 23 to assess the effect of washing treatments statistically. All data were initially tested for normality using Shapiro–Wilk’s test for normality [36] and were normally distributed (*p* ≥ 0.05). Significant mean differences were calculated at (*p* ≤ 0.05) using post hoc Tukey’s HSD test.

The first-order kinetic model was used to describe the dissipation rate of bifenthrin residues in grape and grape leaf samples using the exponential formula of C_t_ = C_o_ e^−kt^, where C_t_ represents the concentration in mg/kg at time t (days), C_o_ represents the concentration at 0 days, and k represents the degradation rate constant (days 1). The half-life was determined using the Hoskins formula: t_1/2_ = In2/k [37]; the pre-harvest interval (PHI) was determined by the formula PHI = Ln (MRL/C_0_)/k [38,39].

## 4. Conclusions

The QuEChERS method was optimized to determine bifenthrin residues in grape leaves. The sample size was reduced to only 5 g, and 5 mL water was added before extraction with acetonitrile to decrease the co-extracted materials in leaf samples. A mixture of adsorbents followed by extract dilution was utilized for cleanup to minimize the matrix effects. The optimized method determined bifenthrin residues in real samples generated from field and laboratory trials. Dissipation of bifenthrin using the normal recommended dose and double the dose in grapevine was also studied in grapes and grape leaves. Dissipation parameters were estimated and discussed. PHI was calculated for grapes and grape leaves in both application doses. Increasing the dose increased the PHI value. The PHI value for leaves was much higher than that of grapes due to the effect of the surface-to-weight ratio. Finally, simple water-washing treatments were applied to reduce residues of bifenthrin. Washing with water effectively reduced bifenthrin residues to the MRL level in grapes and decreased risk to consumers. Washing treatment did not decrease bifenthrin residues to the MRL level in grape leaves.

## Figures and Tables

**Table 1:** Linearity range, correlation coefficient (R^2^), LOD, LOQ, precision, accuracy, and matrix effect of bifenthrin in grape and grape leaves.

	Grape	Grape Leaves
Linearity range (µg/L)	1–100	1–100
R^2^	0.9997	0. 9964
LOD (µg/L)	0.48	0.42
LOQ (µg/kg)	2	20
Intra-day repeatability (RSDr) (n = 6)	5.51 ^a^	6.42 ^b^
Inter-day repeatability (RSD_R_^b^) (n = 18)	7.38 ^a^	6.72 ^b^
Accuracy	98.8	93.5
Matrix effect (%)	−1.17	−3.4

^a^ at 2 µg/kg spiking level (LOQ, the lowest spiking level); ^b^ at 20 µg/kg spiking level (LOQ, the lowest spiking level).

**Table 2:** Dissipation parameters and PHI of bifenthrin in grape and grape leaves.

	Recommended Application Rate		Double Application Rate	
	Grapes	Grapes Leaves	Grapes	Grapes Leaves
Intercept	−0.2542	2.9428	0.1707	3.433
Slope	−0.1549	−0.235	−0.1672	−0.208
R^2 *a*^	0.9873	0.9931	0.9886	0.9919
K *^b^*	0.1549	0.235	0.1672	0.208
t_½_	4.08	1.885	3.54	2.14
MRL *^c^*	0.3	0.01	0.3	0.01
PHI *^d^*	6	32	8	36

*^a^* R^2^ determination coefficient; *^b^* k dissipation rate, days^−1^; *^c^* MRL, mg/kg, EU regulations (EU database, 2024); *^d^* PHI, day value was estimated according to EU-MRL.

**Table 3:** Residue concentration (mg/kg) ±SD of bifenthrin in grape and grapes leaves using washing treatments.

	Grapes	Grapes Leaves
Control, not treated	0.6 ± 0.02 ^C^	10.4 ± 0.69 ^D^
Running water, 1 min	0.4 ± 0.04 ^B^	9.1 ± 0.36 ^C^
Running water, 5 min	0.3 ± 0.05 ^A^	7.9 ± 0.21 ^AB^
Soaking, 1 min	0.4 ± 0.03 ^B^	8.5 ± 0.40 ^BC^
Soaking, 5 min	0.3 ± 0.03 ^A^	6.9 ± 0.08 ^A^

Values with different capital superscripts within the same column are significantly different (*p* ≤ 0.05).

## Data Availability

All data will be available upon reasonable request from the corresponding author.

## Supplementary Materials

The following supporting information can be downloaded at: https://www.mdpi.com/article/10.3390/plants13121695/s1, Table S1. Recovery and RSD % of bifenthrin in grapes and grape leaves. Table S2. Residue data (mg/kg ± SD) of bifenthrin in grape and grape leaves.
