# Supplementary material for: Bifenthrin Residues in Table Grapevine: Method Optimization, Dissipation and Removal of Residues in Grapes and Grape Leaves
Source: Plants (Basel). 2024 Jun 19;13(12):1695. doi: 10.3390/plants13121695 (PMC11207924; doi:10.3390/plants13121695)
Supplement: Supplementary file 1 [file plants-13-01695-s001.zip › plants-3000699-supplementary.pdf]

Table S1. Recovery and RSD % of bifenthrin in grapes and grape leaves

Spiking levels	Grape	Grape leaves
(µg/kg)	Recovery % (RSD, n=6)	Recovery % (RSD, n=6)
10	97.7 (6.48)	-
50	-	90.9 (5.63)
100	100.7 (5.81)	96.4 (2.97)
1000	98.1 (1.73)	93.1 (6.27)
Average recovery %	98.8 (4.7)	93.5 (4.9)

Table S2. Residue data (mg/kg ±SD) of bifenthrin in grape and grape leaves

Time (days)	Recommended application rate		Double application rate	
	Grapes	Grapes leaves	Grapes	Grapes leaves
0	0.82±0.43	24.36±9.85	1.31±0.69	39.67±7.88
1	0.61±0.32	14.57±3.69	0.99±0.53	22.57±8.97
3	0.43±0.17	9.74±2.76	0.66±0.23	16.18±6.48
7	0.29±0.14	3.69±1.16	0.39±0.13	8.89±3.56
10	0.17±0.11	1.42±0.59	0.22±0.19	3.12±1.13
14	0.08±0.08	0.64±0.33	0.09±0.03	1.39±0.99
20	0.05±0.04	0.12±0.09	0.06±0.04	0.44±0.25
24	0.02±0.02	0.09±0.04	0.02±0.04	0.19±0.22
28	0.01±0.01	0.03±0.02	0.01±0.02	0.12±0.17
